# Dynamic changes in choroidal conditions during anti-vascular endothelial growth factor therapy in polypoidal choroidal vasculopathy

**DOI:** 10.1038/s41598-019-47738-9

**Published:** 2019-08-06

**Authors:** Norihiro Nagai, Misa Suzuki, Sakiko Minami, Toshihide Kurihara, Mamoru Kamoshita, Hideki Sonobe, Kazuhiro Watanabe, Atsuro Uchida, Hajime Shinoda, Kazuo Tsubota, Yoko Ozawa

**Affiliations:** 1 Laboratory of Retinal Cell Biology, Keio University, School of Medicine, Tokyo, Japan; 2 Department of Ophthalmology, Keio University, School of Medicine, Tokyo, Japan

**Keywords:** Risk factors, Retinal diseases

## Abstract

We defined the relationships between initial choroidal conditions and their dynamics and exudative changes during anti-vascular endothelial growth factor (anti-VEGF) therapy in polypoidal choroidal vasculopathy (PCV). One hundred treatment-naïve eyes of 100 patients with PCV treated for 24 months at Keio University Hospital with intravitreal ranibizumab or aflibercept monotherapy (three injections and PRN thereafter) were retrospectively analyzed. Wet macula risk after three induction injections, which affected visual prognosis, was predicted by initial pachyvessels in the choroid (foveal greatest vertical choroidal vessel diameter [CVD] ≥180 μm) and pachychoroid (central choroidal thickness [CCT] ≥220 μm) recorded by optical coherence tomography. The risk for recurrent exudative change was greater in the pachyvessel groups irrespective of presence or absence of pachychoroid. Mean CVD and CCT decreased with anti-VEGF therapy when achieving a dry macula, suggesting that exudative changes are regulated by VEGF. Mean CVD and CCT at remission were greater in patients with initial pachyvessels and pachychoroid than in those without; the basal levels of CVD and CCT most likely represent VEGF-unrelated conditions. CVD increase preceded CCT increase and recurrent exudative changes, suggesting that the VEGF-related CVD increase may regulate CCT and exudative change; and that CVD may be a biomarker of exudative change.

## Introduction

Polypoidal choroidal vasculopathy (PCV), as defined by Yannuzzi *et al*^1^., is characterized by polypoidal lesions and branching vascular networks with recurrent serosanguinous maculopathy^2^ and is prevalent among Asian and African-American populations^3,4^. It is included as a subtype of age-related macular degeneration (AMD) with a substantially shared genetic susceptibility with typical AMD^5^ and has been successfully treated using anti-vascular endothelial growth factor (anti-VEGF) drugs— both in clinical trials^6–9^ and in real-world clinical settings^10–12^. In addition, photodynamic therapy (PDT), with or without anti-VEGF drugs, has been reviewed and studied in multicenter randomized clinical trials^13,14^. However, we and others have previously reported that treatment response and clinical prognosis are different according to the number of polypoidal lesions present^15^, or lesion size^16,17^, or genetic background^18^. Therefore, PCV exhibits heterogeneity in treatment responses^15–19^.

One explanation for the heterogeneity may be the choroidal condition, which can be assessed by measuring the central choroidal thickness (CCT) and/or choroidal vessel diameter (CVD). The concept of pachychoroid spectrum diseases was proposed by Freund *et al*.^2,20,21^ the spectrum of diseases is associated with choroidal thickening and includes PCV as well as pachychoroid pigment epitheliopathy, central serous chorioretinopathy, and type 1 pachychoroid neovasculopathy^20–22^. Miyake *et al*. reported that patients with neovascularization in the choroid with a CCT ≥200 μm, called pachychoroid neovasculopathy, had lower genetic risk scores of susceptibility to neovascular AMD without pachychoroid^23^, and proposed a difference in etiology depending on the presence or absence of pachychoroid. Regarding PCV, Koizumi *et al*. reported that the mean CCT was thicker in patients with PCV than in those with typical AMD as a whole^24^; although, there was a pronounced inter-individual variability with CCT values^25^. In addition, Lee *et al*. reported that pachyvessels (larger diameter blood vessels) in Haller’s layer (the outermost layer of the choroid) were identified under the site of neovascular ingrowth in more than 90% of the PCV eyes with a thin choroid (CCT ≤200 μm)^25^, indicating that there were PCV patients who had pachyvessels in the choroid with no thickening of the choroid. Thus, the impact of choroidal conditions on the pathogenesis and the relationships between pachyvessels and pachychoroid are not fully understood. Moreover, dynamic changes in CVD and CCT, during clinical course of treating exudative changes, have not been analyzed. In addition, Cheung *et al*. described in their review article that criteria and the definitive threshold for pachychoroid and pachyvessels have not been developed and may be determined by characteristic morphological changes, which alter choroidal function^2^. Meanwhile, the cut-off values based on differences in treatment responses and recurrences would be useful in evaluating prognosis in the clinic and analyzing whether there may be clinical differences in PCVs according to choroidal conditions.

In this study, we evaluated whether responses to anti-VEGF therapy differ according to choroidal conditions in the patients who were diagnosed with PCV by focusing on the achievement of a dry macula after three initial monthly injections as the induction phase and the prognosis after 24 months of follow-up. The cut-off values of the choroidal conditions that showed different clinical courses were analyzed. We then demonstrated dynamic choroidal changes over time during anti-VEGF monotherapy using a pro re nata (PRN) regimen following the induction phase and compared the values of CVD and CCT at respective time points of remission and recurrence among groups, which were divided based on whether the patients had pachyvessels and pachychoroid at baseline, to discuss differences in the clinical course of PCVs with or without pachyvessels and/or pachychoroid and the mechanism of the pachychoroid spectrum.

## Results

### Baseline characteristics and overall outcomes

**Figure 1:**
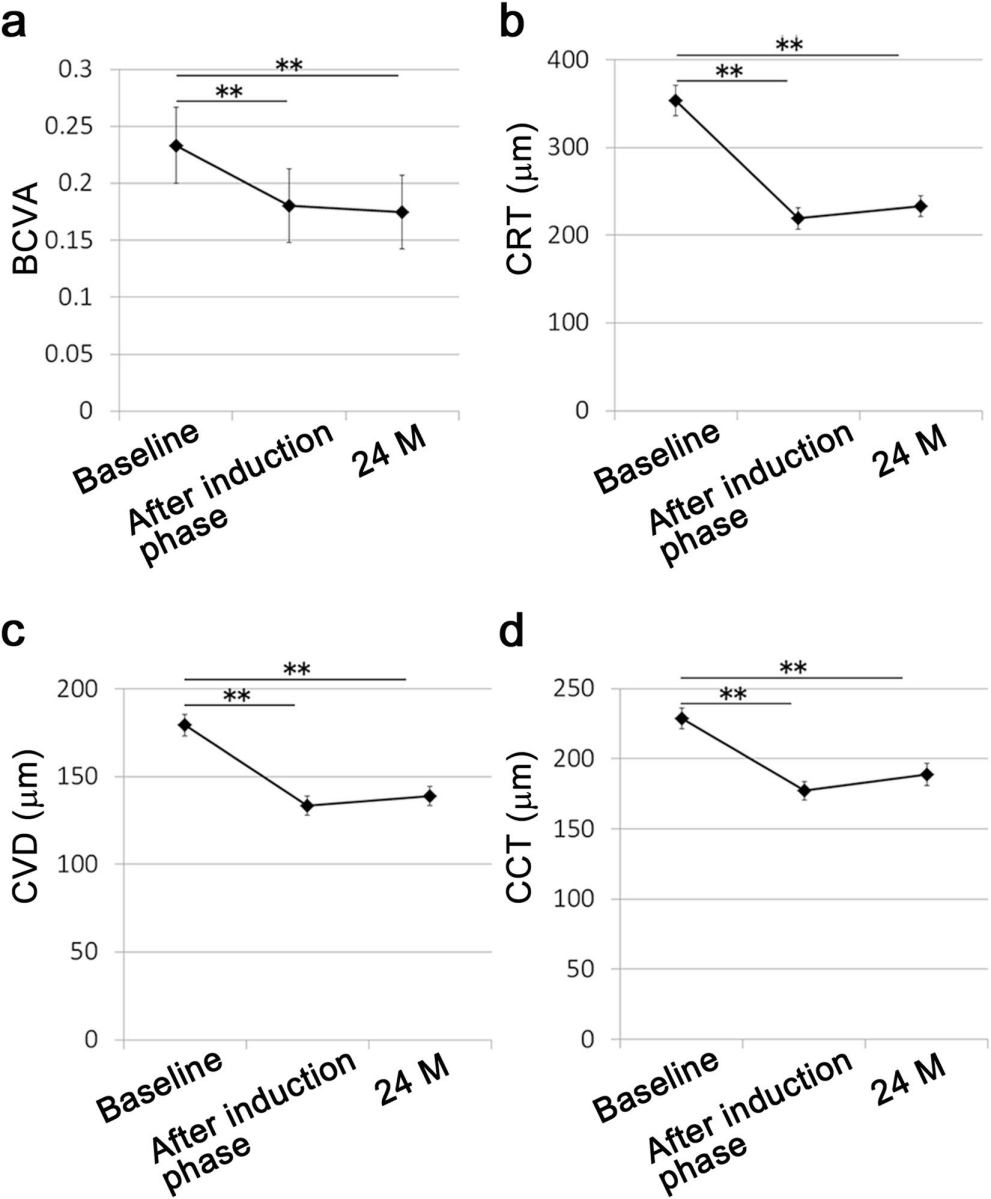
Mean outcome values over time during anti-vascular endothelial growth factor therapy. A two-tailed *t*-test was performed between the data at baseline and after the induction phase or at 24 months. Mean best-corrected visual acuity (BCVA) (**a**), central retinal thickness (CRT) (**b**), choroidal vessel diameter (CVD) (**c**), and central choroidal thickness (CCT) (**d**) were significantly improved after the induction phase and at 24 months. Data are shown as the mean ± SE. **P < 0.01.

Among 100 eyes of 100 patients (69.4 ± 0.9 years of age), 70 (70%) belonged to men (Table 1). Overall, the mean best-corrected visual acuity (BCVA) (Fig. 1a), central retinal thickness (CRT) (Fig. 1b), vertical CVD at the foveal region (Fig. 1c), and CCT (Fig. 1d) measured in the OCT images were significantly improved after the induction phase compared with the initial values, and the improvement was preserved at month 24 following the PRN regimen. During the 24-month follow-up, the mean number of injections was 7.4 ± 0.4. There were no differences in the data at baseline and at 24 months between the eyes that underwent intravitreal ranibizumab (IVR) and aflibercept (IVA) injections (data not shown).

**Table 1:** Baseline characteristics.

Age	69.4 ± 0.9
Gender (male; eyes [%])	70 (70.0)
BCVA (LogMAR)	0.236 ± 0.033
CRT (μm)	354 ± 17
CVD (μm)	179 ± 6
CCT (μm)	228 ± 7
**Fundus findings**	
Intraretinal fluid (eyes [%])	19 (19.0)
Serous retinal detachment (eyes [%])	75 (75.0)
Retinal hemorrhage (eyes [%])	46 (46.0)
Serous PED (>2DD) (eyes [%])	18 (18.0)
Hemorrhagic PED (>3DD) (eyes [%])	12 (12.0)
Multiple polyps (eyes [%])	30 (30.0)

Data are shown in mean ± SE. BCVA, best and on recurrences during 24 months of follow-up-corrected visual acuity; CRT, central retinal thickness; CVD, choroidal vessel diameter; CCT, central choroidal thickness; PED, pigment epithelial detachment; DD, disc diameter.

### Treatment outcomes of patients with a still-wet macula after the induction phase

After three monthly injections of anti-VEGF drugs in the induction phase, 31 eyes (31%) still exhibited exudative changes above the retinal pigment epithelium (RPE) and/or hemorrhage at any level in the fundus, and thus had a wet macula (Table 2). The mean BCVA was worse and the mean CRT was greater at 24 months in the 31 eyes, although the initial BCVA and CRT were not different between the two groups (data not shown). The injections in these 31 eyes were significantly more frequent in average than in the eyes with a dry macula after the induction phase (Table 2). Thus, the eyes with a wet macula after the induction phase had a worse prognosis than those with a dry macula.

**Table 2:** Twenty-four months outcomes of the patients with a wet or dry macula after induction phase.

Eyes	Wet macula	Dry macula	P value
	31	69	
**Data at month 24**			
BCVA (LogMAR)	0.285 ± 0.075	0.128 ± 0.003	0.023*
CRT (μm)	269 ± 28	217 ± 11	0.048*
CVD (μm)	140 ± 9	138 ± 6	0.862
CCT (μm)	182 ± 15	200 ± 9	0.334
Mean injection number	8.7 ± 0.7	6.8 ± 0.4	0.024*
History of recurrences (eyes [%])	19 (62.3)	58 (79.3)	0.077

Data are shown in mean ± SE. A two-tailed *t*-test or Chi-squared test was performed. BCVA, best-corrected visual acuity; CRT, central retinal thickness; CVD, choroidal vessel diameter; CCT, central choroidal thickness. *P < 0.05, **P < 0.01.

### Predictors of a wet macula after the induction phase

Subsequently, predictors of a wet macula following the induction phase were evaluated by multivariable logistic regression analyses adjusting for age and gender (Table 3). When we define the pachyvessels as CVD ≥180 μm and pachychoroid as CCT ≥220 μm, we found that eyes having initial pachyvessels (OR 5.834 [95%CI 2.053 to 16.57]; P = 0.008) and pachychoroid (OR 4.970 [95%CI 1.840 to 13.43]; P = 0.002) had respective significant risks of wet macula following the induction phase. In these definitions, the ORs were the greatest with significant risks.

**Table 3:** Predictors of wet macula after induction phase.

	OR	95%CI	P value
Pachyvessels in the choroid (CVD ≥180 μm)	5.834	2.053 to 16.57	0.008**
Pachychoroid (CCT ≥220 μm)	4.970	1.840 to 13.43	0.002**

Multivariable logistic regression analyses adjusted for age and gender. CVD, choroidal vessel diameter; CCT, central choroidal thickness. **P < 0.01.

### Risks for exudative changes in the patients with a CVD ≥180 μm and/or CCT ≥220 μm at baseline

To compare the risk of exudative changes with or without pachyvessels and pachychoroid, the patients were divided into four groups according to the initial vertical CVD (thin vessels [CVD <180 μm] or pachyvessels [CVD ≥180 μm] in the choroid) and initial CCT (thin choroid [CCT <220 μm] or pachychoroid [CCT ≥220 μm]) (Table 4). The mean age was lower in the groups with pachyvessels with or without pachychoroid (P < 0.05). In the eyes presenting with both initial pachyvessels in the choroid and pachychoroid, a greater number of the eyes had a wet macula after the induction phase (OR 8.79 [95%CI 1.27 to 3.16]; P = 0.003) and at 24 months (OR 4.03 [95%CI 1.17 to 13.9]; P = 0.007) after adjusting for age and gender.

**Table 4:** Exudative changes during anti-vascular endothelial growth factor monotherapy without or with pachyvessels in the choroid and/or pachychoroid.

Initial CVD	<180 μm	<180 μm	≥180 μm	≥180 μm
Initial CCT	<220 μm	≥220 μm	<220 μm	≥220 μm
Eyes	29	20	18	33
Age (mean ± SE)	72.8 ± 1.1	71.1 ± 2.4	66.9 ± 2.7	66.6 ± 1.4
Gender (Male [%])	18 (62.7)	14 (70.0)	12 (66.7)	26 (78.8)
**Wet macula**				
After induction phase (eyes [%])	4 (13.8)	6 (30.0)	3 (16.7)	18 (54.5)
Odds ratio (P, 95%CI)	1	1.69 (0.16, 0.82–3.51)	1.71 (0.56, 0.27–10.6)	**8**.**79** (**0**.003**, **1**.**27–3**.**16**)
At 24 M (eyes [%])	8 (27.6)	5 (25.0)	4 (22.2)	17 (51.5)
Odds ratio (P, 95%CI)	1	0.86 (0.82, 0.23–3.20)	1.04 (0.96, 0.24–4.47)	**4**.**03** (**0**.03*, **1**.**17–13**.**9**)
Recurrences (eyes [%])	16 (51.7)	17 (85.0)	15 (83.3)	27 (81.8)
Odds ratio (P, 95%CI)	1	1.79 (0.10, 0.90–3.54)	**5**.**99** (**0**.04*, **1**.**12–31**.**9**)	**7**.**17** (**0**.01*, **1**.**49–34**.**5**)
Injection number (mean ± SE)	6.0 ± 0.6	8.8 ± 1.0	7.8 ± 1.0	6.7 ± 0.5

A one-way analysis of variance (ANOVA) with Turkey’s post hoc test and multivariable logistic regression analyses adjusted for age and gender at the time of initial injection. CCT, central choroidal thickness; CVD, choroidal vessel diameter. *P < 0.05, **P < 0.01.

In the 97 eyes that achieved a dry macula during the 24 month period,excluding 3 eyes that did not achieve a dry macula despite receiving constant injections, 75 eyes (77.3%) exhibited recurrent exudative changes including hemorrhage after transient remission within the 24-month period (data not shown). After adjusting for age and gender, groups exhibiting initial pachyvessels with (OR 7.17 [95%CI 1.49 to 34.5]; P = 0.01) or without (OR 5.99 [95%CI 1.12 to 31.9]; P = 0.04) pachychoroid had a greater risk. The three eyes that persistently had a wet macula were in the group with the initial pachyvessels and pachychoroid.

### Dynamic changes in choroidal conditions at the time of the first remission and recurrence

**Figure 2:**
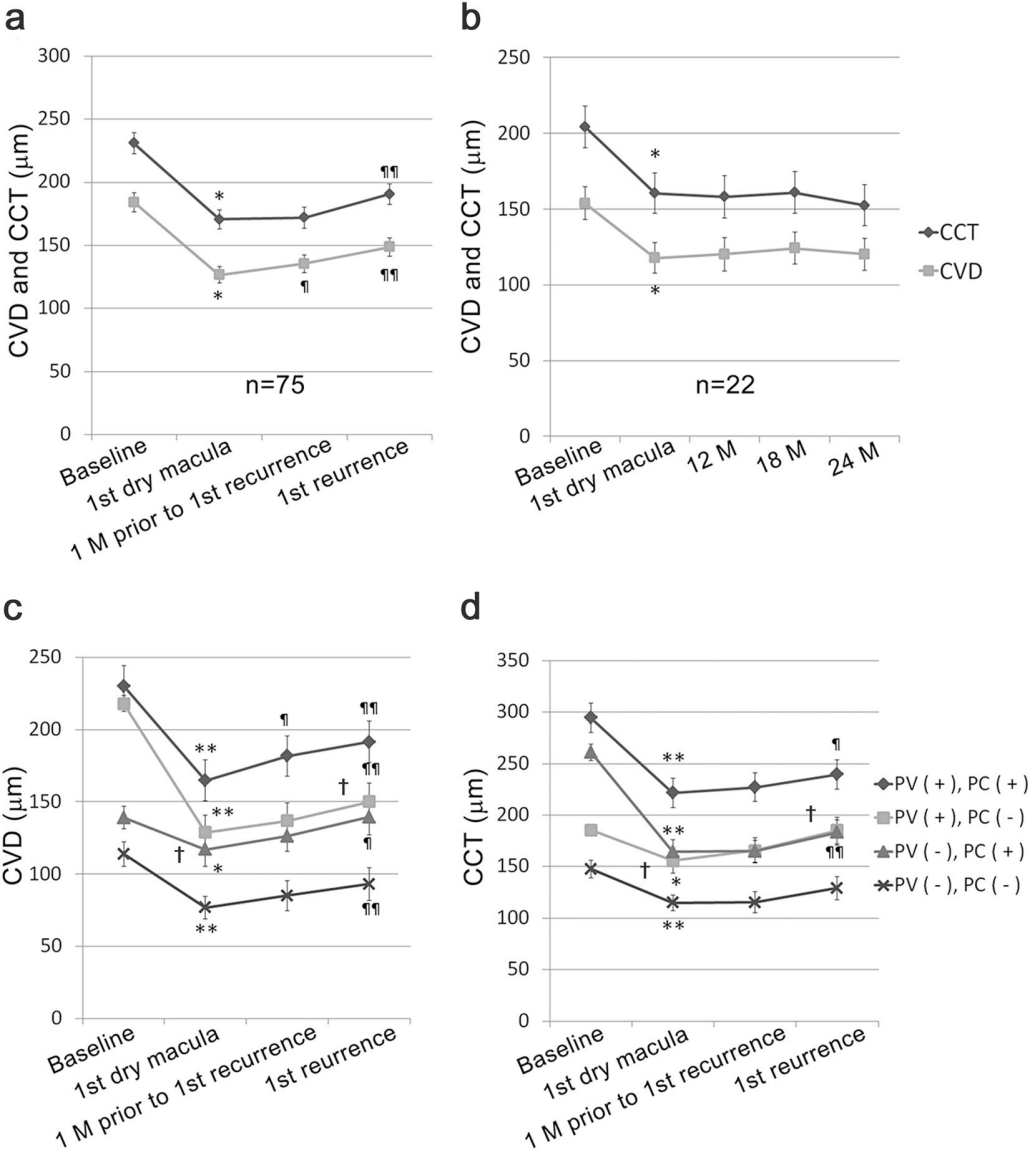
Dynamic changes in mean vertical choroidal vessel diameter (CVD) and central choroidal thickness (CCT) after anti-vascular endothelial growth factor (anti-VEGF) therapy. A two-tailed *t*-test was performed between the data at baseline and at the time of achieving the first dry macula (*P < 0.05, **P < 0.01), between the time of achieving the first dry macula and the indicated later time points (¶P < 0.05, ¶¶P < 0.01), and between the data of pachyvessel (+) and pachychoroid (+) group and the pachyvessel (−) and pachychoroid (−) group at respective time points of remission and recurrence (^†^P < 0.001). (**a**) Mean CVD and CCT of the patients who had recurrent exudative changes (n = 75). The mean CVD and CCT were significantly decreased at the time of achieving the first dry macula after anti-VEGF treatment. Compared with the time of the first dry macula, the mean CVD and CCT were increased at the time of the first recurrence. In addition, the mean CVD was already increased one month prior to recurrence. (**b**) Mean CVD and CCT changes in the patients who had no recurrent exudative changes after achieving a dry macula until 24 months (n = 22). Compared with the baseline, the mean CVD and CCT were significantly decreased at the time of achieving a dry macula (*P < 0.05), and the values were not changed until 24 months. (**c**,**d**) The recurrent patients were divided into four groups with or without pachyvessels in the choroid (CVD ≥180 μm) and pachychoroid (CCT ≥220 μm). Compared with the baseline, the mean CVD (**c**) and CCT (**d**) were significantly decreased at the time of achieving the first dry macula after anti-VEGF treatment in all groups. (**c**) The mean CVD was increased at the time of the first recurrence in all groups. A CVD increase one month prior to the first recurrence was significant in the eyes with pachyvessels in the choroid and pachychoroid. (**d**) The mean CCT increase at the time of the first recurrence of exudative changes was significant in the eyes with pachyvessels with and without pachychoroid. (**c**,**d**) The mean CVD and CCT at the times of remission and recurrence were significantly different between the PV+, PC+ group and the PV−, PC− group. PV, pachyvessels in the choroid; PC, pachychoroid. n = 27 (PV+, PC+), 15 (PV+, PC−), 17 (PV−, PC+), 16 (PV−, PC−). Data are shown as the mean ± SE.

To elucidate whether choroidal changes are involved in the pathogenesis of PCV-related exudative changes, CVD and CCT at the time of achieving the first dry macula, one month prior to the first recurrence, and the first recurrence were analyzed in the eyes with recurrence (Fig. 2a). The mean CVD and CCT were both reduced at the time of the first remission achieving a dry macula; however, they were increased at the time of the first recurrence. Moreover, an increase in the mean CVD preceded the recurrent exudative changes; the mean CVD was already increased one month prior to the recurrence. In contrast, the other 22 eyes that did not have recurrences after the first remission showed a decreased mean CVD and CCT at the time of achieving a dry macula and did not show changes in the values throughout the rest of the course (Fig. 2b).

The changes in CVD (Fig. 2c) and CCT (Fig. 2d) were compared in the four groups, which were divided according to the initial CVD and CCT values. In all the groups, both the CVD and CCT were significantly decreased at remission (P < 0.05 or <0.01), and the CVD was increased at the time of recurrence (P < 0.01 in all). The significant increase in CVD one month prior to the first recurrence was obvious in the group that initially exhibited both pachyvessels in the choroid and pachychoroid (P < 0.05); a similar trend was also observed in the other groups. The significant increase in CCT at the time of recurrence, as compared with that at remission, was observed only in the groups having pachyvessels (CVD ≥180 μm) irrespective of the presence (P < 0.05) or absence (P < 0.01) of pachychoroid.

Each value of CVD (p < 0.001) and CCT (p < 0.001) at the times of remission and recurrence was significantly different between the pachyvessel (+) and pachychoroid (+) group and the pachyvessel (−) and pachychoroid (−) group (Fig. 2c,d, Supplementary Table S1).

## Discussion

We demonstrated that the presence of pachyvessels in the choroid and pachychoroid, as defined by a vertical CVD **≥**180 μm and CCT ≥220 μm at baseline, respectively, were risk factors for a still-wet macula after the three monthly injections during the induction phase, which reflected the worse visual outcome at 24 months. Among the four groups, which were divided based on whether they had pachyvessels in the choroid and/or pachychoroid at baseline, the recurrence risk was higher in the groups having pachyvessels with or without pachychoroid. The mean CVD and CCT were decreased at the time of achieving a dry macula and increased at the time of recurrent exudative changes in all groups. Moreover, an increase in CVD was detected one month prior to the first recurrence; this was overall significant and particularly pronounced in the pachyvessels with CVD ≥180 μm (+) and pachychoroid with CCT ≥220 μm (+) group. Interestingly, the CVD and CCT not only at the time of recurrence but remission differed based on whether the eyes had pachyvessels in the choroid and pachychoroid at baseline.

Overall visual prognosis, CRT, CVD, and CCT were improved following treatment in the current study; the mean CCT was reduced by 9% at month 24 (data not shown), which is consistent with the results of a previous report showing a 13% reduction in CCT at month 12 with bimonthly aflibercept treatment after the three induction injections in a sample of eyes with exudative AMD, including PCV and typical AMD^26^. A dry macula was achieved with the induction treatment in 69% of the treatment-naïve eyes in the current study, which is consistent with the results of previous studies on eyes with PCV and typical AMD; it was achieved in 71.6% of the treatment naïve eyes^27^ and in 68.8% of eyes with or without previous treatments^28^ after the three monthly injections of aflibercept and ranibizumab, respectively. The recurrence rate is reported to be as high as 40 to 80% by 3 years^2,29–31^, and the current result, 77.3%, was within this previously reported range.

Interestingly, a still-wet macula after three monthly anti-VEGF injections was predicted by pachyvessels in the choroid and pachychoroid as defined by a CVD ≥180 μm, and CCT ≥220 μm, respectively. Recurrence after achieving a dry macula was also related to pachyvessels with a CVD ≥180 μm at baseline. Evaluating CVD and CCT will help patients and physicians estimate the prognosis and plan a treatment schedule at an early date in the clinic.

We then focused on the dynamic changes in choroidal conditions during anti-VEGF therapy. In this study, which involved PRN treatment, CCT was reduced with the anti-VEGF therapy at the time of remission, findings similar to those of previous studies^12,24,27^. Moreover, CVD was also reduced in the current study. The reductions were still obvious after dividing the eyes into four groups based on the initial CVD and CCT values. Both the CVD and CCT were increased at the time of the first recurrence compared with the overall values at the time of remission. Considering that a CVD increase preceded the recurrence, especially in the group exhibiting both pachyvessels and pachychoroid, and that the odds ratio of recurrence was similar among patients with pachyvessels in the choroid irrespective of the presence of pachychoroid, CVD rather than CCT may be responsible for exudative changes in PCV. These results suggest that the CVD increase induced a CCT increase and recurrent exudative changes. Thus, CVD may be an indicator and a biomarker of disease activity.

Strikingly, both the CVD and CCT at the time of achieving the first dry macula were significantly different between the group with both the pachyvessels in the choroid and pachychoroid and the group with neither. The anti-VEGF therapy did not lower the CVD or CCT in the pachyvessel (+) and pachychoroid (+) group to the degree seen in the pachyvessel (−) and pachychoroid (−) group, suggesting that the pachyvessels and pachychoroid before the activation of the lesion and the onset of exudative changes may involve mechanisms that are not VEGF-related. Thus, CVD and CCT may not be necessarily regulated only by VEGF in pachyvessel (+) and pachychoroid (+) eyes. Lee *et al*.^32^ and Hata *et al*.^33^ reported that the intraocular VEGF level was lower in patients with pachychoroid, supporting the idea. The mechanism may involve other inflammatory cytokines. Alternatively, the balance of sympathetic and parasympathetic activities for autoregulation of choroidal flow may be disturbed, given that choroidal flow is regulated mainly by a neurogenic mechanism rather than hypoxia/normoxia^34^. The patients with pachyvessels with a CVD ≥180 μm were significantly younger and may have had different genetic backgrounds^23,35^. In contrast, an increase in CVD and CCT resulting in recurrence may be regulated by VEGF, given that anti-VEGF therapy may effectively reverse the change.

Both the CVD and CCT at the time of recurrence were also significantly greater in the pachyvessel (+) and pachychoroid (+) group than in the pachyvessel (−) and pachychoroid (−) group, suggesting that the threshold of the RPE integrity to avoid exudative changes might be higher in the pachyvessel (+) and pachychoroid (+) group; this may also be related to the fact that the patients were younger in the pachyvessel (+) and pachychoroid (+) group and that the aging of and/or damage to the RPE was not as severe when compared with PCV patients without pachy-phenotypes.

Other than CVD and CCT, choroidal vascular hyperpermeability (CVH) may also be of note in pachychoroid spectrum diseases^36–39^, which is also observed in the asymptomatic fellow eyes^40^. The relationship between pachychoroid and CVH and its impact on the pathogenesis of PCV have recently been analyzed^40–42^. The relationship between changes in CVD and CVH would be an interesting topic for future study, although it is difficult to quantify CVH.

While PCV and the pachychoroid condition may be frequently seen in the Asian population^4^, the current data also show dynamic changes in the choroidal conditions of patients with thin vessels and/or a thin choroid. The choroidal vascular dilation and thickening were related to the exudative change, and the dynamic change was related to VEGF, irrespective of the presence of pachyvessels and pachychoroid. Thus, the results fit the global interest and are also relevant to the other racial cohorts; additionally, the results may facilitate the future study of dynamic changes in the choroidal condition in other subtypes of AMD. Furthermore, the basal level of the choroidal condition may be regulated by a mechanism that is not VEGF-related; however, exudative related dynamic choroidal changes were VEGF-related. Thus, the concept that two different regulatory mechanisms exist in the choroid may help deepen our understanding of choroidal diseases and necessitate further studies in the future.

There were limitations in the current study, which included its retrospective design and relatively small sample size. Re-injection during the PRN regimen was determined by individual physicians and not the reading center; additionally, the data included those of patients treated with either aflibercept or ranibizumab. However, OCT images and fundus photographs clearly indicated the presence or absence of exudative changes, including hemorrhage during the course, and appropriateness of the re-injections were retrospectively confirmed. Including patients treated by either drug may underestimate the reduction in the CVD and/or CCT based on differences in the extent of VEGF suppression according to the drug type. Nonetheless, we obtained significant data. The point also enabled us to demonstrate the generality of the results during anti-VEGF therapy.

In summary, the initial presence of pachyvessels in the choroid (vertical CVD ≥180 μm) and pachychoroid (CCT ≥220 μm) were risk factors for the exudative changes remaining after the induction phase; additionally, pachyvessels in the choroid were a risk factor for recurrence with or without pachychoroid in PCV. Both the CVD and CCT decreased at the time of achieving a dry macula in response to anti-VEGF therapy and increased when the eye had recurrent exudative changes in all the groups, whereas a CVD increase preceded the recurrence. Taken together, dynamic changes in the CVD may regulate choroidal thickness to induce exudative changes in PCV, and the disease activity may be regulated by VEGF. Choroidal conditions, in particular dynamic CVD change, could be a biomarker of recurrent exudative changes, whereas the initial CVD may determine the risk of recurrence. The CVD and CCT differed between the groups with or without pachyvessels and pachychoroid even at the time of achieving a dry macula after anti-VEGF therapy, suggesting that in the pachychoroid spectrum groups, regulatory mechanisms that are not VEGF-related may be involved in the basal choroidal conditions. Thus, the difference in background choroidal condition may cause different clinical courses during anti-VEGF therapy in PCV, although further study is required.

## Methods

The current study adhered to the tenets of the Declaration of Helsinki, was approved by the Ethics Committee of the Keio University School of Medicine (Tokyo, Japan; 2010002), and was registered as UMIN000007649. Informed consent was obtained from all subjects.

### Subjects

This was a retrospective study based on the medical chart review of 100 eyes in 100 patients who were diagnosed with PCV and unilaterally treated with either intravitreal ranibizumab (IVR) or aflibercept (IVA) monotherapy at the Medical Retina Division Clinic (AMD Clinic) of the Department of Ophthalmology, Keio University Hospital between June 2009 and April 2016. All patients attended the clinic for at least 24 months. Patients who underwent surgery or PDT or other drug injections or who did not receive re-injection in accordance with the re-injection criteria during the course were excluded.

### Ocular examinations

All subjects underwent BCVA measurements with refraction testing throughout the course of treatment, slit-lamp examination, and binocular indirect ophthalmoscopy after pupil dilation with 0.5% tropicamide. These examinations were performed at every follow-up visit.

### Angiography

Fluorescein angiography (FA) and indocyanine green angiography (IA) were performed at baseline using a retinal camera (TRC50DX, Topcon, Tokyo, Japan). Diagnoses of PCV were made according to the Treatment of Age-Related Macular Degeneration with Photodynamic Therapy criteria and performed by retina specialists (NN, MS, and YO)^43^.

### Optical coherence tomography

**Figure 3:**
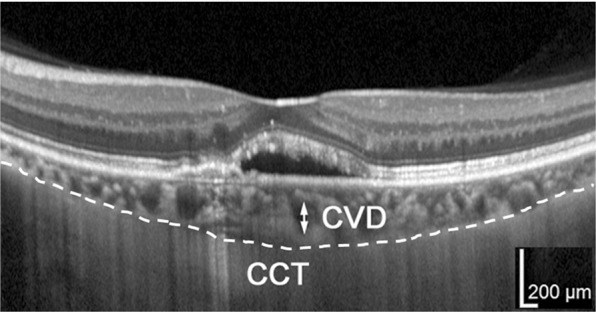
Measurement of choroidal vessel diameter (CVD) and central choroidal thickness (CCT) in the optical coherence tomography (OCT) image. The CVD (vertical diameter of the thickest outer choroidal vessel in the foveal region, arrow) and CCT were measured by referring to the scale bars in the OCT system. Choroid is contoured by the dotted line.

Optical coherence tomography (OCT) images were recorded at every follow-up visit using an OCT instrument (Spectralis, Heidelberg Engineering, Dossenheim, Germany). CRT was defined as the distance between the internal limiting membrane and the presumed RPE at the fovea. The vertical diameter of the thickest outer choroidal vessel in Haller’s layer of the foveal region was defined as the CVD (Fig. 3). The CRT, CVD, and CCT were measured by referring to the scale bars in the OCT system. Dry macula was defined as having no fluid above the RPE and no hemorrhage in the fundus.

### Intravitreal injections

Either ranibizumab (0.5 mg [0.05 mL]) or aflibercept (2 mg [0.05 mL]) were injected intravitreally under sterile conditions via the pars plana once per month for 3 months as the induction phase, and then as needed (PRN). Any intra- and/or subretinal fluid and hemorrhage in the fundus at the time of the visits was re-treated.

### Statistical analyses

Commercially available software (SPSS version 23.0, IBM Corporation, Armonk, NY, USA) was used for statistical analysis; P < 0.05 was considered statistically significant. Two-tailed t-tests, the chi-squared test, Pearson product-moment correlation coefficient, one-way analysis of variance with Tukey’s post hoc testing, and multivariable logistic regression analyses were performed. All results are expressed as the mean ± standard error.

## Supplementary information

Supplementary Table S1

## Data Availability

The protocol and the datasets generated during and/or analyzed during the current study are available from the corresponding author upon reasonable request.
