# Supplementary material for: Dynamic changes in choroidal conditions during anti-vascular endothelial growth factor therapy in polypoidal choroidal vasculopathy
Source: Sci Rep. 2019 Aug 6;9:11389. doi: 10.1038/s41598-019-47738-9 (PMC6684594; doi:10.1038/s41598-019-47738-9)
Supplement: Supplementary file 1 — Supplementary Table S1 [file 41598_2019_47738_MOESM1_ESM.pdf]

Dynamic changes in choroidal conditions during anti-vascular endothelial growth factor therapy in polypoidal choroidal vasculopathy

Norihiro Nagai 1a,1b, Misa Suzuki 1a,1b, Sakiko Minami 1b, Toshihide Kurihara 1b, Mamoru Kamoshita 1a,1b, Hideki Sonobe 1b, Kazuhiro Watanabe 1b, Atsuro Uchida 1b, Hajime Shinoda 1b, Kazuo Tsubota 1b, Yoko Ozawa* 1a,1b

1a Laboratory of Retinal Cell Biology, 1b Department of Ophthalmology, Keio University, School of Medicine

***Corresponding author:**

Yoko Ozawa, M.D., Ph.D.

Laboratory of Retinal Cell Biology

Department of Ophthalmology

Keio University School of Medicine

35 Shinanomachi, Shinjuku-ku, Tokyo 160-8582, Japan

Phone: +81-3-5363-3869, Fax: +81-3-5363-3869

E-mail: ozawa@a5.keio.jp

Supplementary Table S1. Dynamic choroidal changes in recurrent eyes

**Initial CVD**	**<180 μm**	**<180 μm**	**≥180 μm**	**≥180 μm**	
**Initial CCT**	**<220 μm**	**≥220 μm**	**<220 μm**	**≥220 μm**	**P value**
Eyes	16	17	15	27	
**CVD (μm)**					
At baseline	114 ± 11	144 ± 9	218 ± 10	230 ± 9	<0.001**
At 1st dry macula	77 ± 7	107 ± 12	129 ± 12	165 ± 10	<0.001**
1 M prior to recurrence	85 ± 9	119 ± 11	137 ± 11	182 ± 10	<0.001**
At recurrence	93 ± 11	130 ± 13	150 ± 11	191 ± 11	<0.001**
**CCT (μm)**					
At baseline	144 ± 9	260 ± 7	187 ± 5	295 ± 11	<0.001**
At 1st dry macula	115 ± 8	161 ± 16	156 ± 13	222 ± 11	<0.001**
1 M prior to recurrence	115 ± 10	161 ± 17	166 ± 13	227 ± 11	<0.001**
At recurrence	129 ± 11	177 ± 17	185 ± 13	240 ± 11	<0.001**

Data are shown mean ± SE. A one-way analysis of variance (ANOVA) with Turkey's post hoc test and multivariable logistic regression analyses adjusted for age and gender at the time of initial injection. CCT, central choroidal thickness; CVD, choroidal vessel diameter. P value of greater CCT and CVD group compared with smaller CCT and CVD group. *P<0.05, **P<0.01.
